# Optimization of whole-brain rabies virus tracing technology for small cell populations

**DOI:** 10.1038/s41598-021-89862-5

**Published:** 2021-05-17

**Authors:** Theresia J. M. Roelofs, Shanice Menting-Henry, Lieke M. Gol, Annelijn M. Speel, Vera H. Wielenga, Keith M. Garner, Mieneke C. M. Luijendijk, Alexandru A. Hennrich, Karl-Klaus Conzelmann, Roger A. H. Adan

**Affiliations:** 1 Department of Translational Neuroscience, Brain Center Rudolf Magnus, University Medical Center Utrecht, Universiteitsweg 100, 3584 CG Utrecht, The Netherlands; 2 Biomedical MR Imaging and Spectroscopy Group, Center for Image Sciences, University Medical Center Utrecht and Utrecht University, Bolognalaan 50, 3584 CJ Utrecht, The Netherlands; 3 Institute of Neuroscience and Physiology, The Sahlgrenska Academy At the University of Gothenburg, Gothenburg, Sweden; 4 Max Von Pettenkofer Institute Virology and Gene Center, Medical Faculty, Ludwig-Maximilians-University Munich, 81377 Munich, Germany

**Keywords:** Molecular biology, Neuroscience

## Abstract

The lateral hypothalamus (LH) is critically involved in the regulation of homeostatic energy balance. Some neurons in the LH express receptors for leptin (LepRb), a hormone known to increase energy expenditure and decrease energy intake. However, the neuroanatomical inputs to LepRb-expressing LH neurons remain unknown. We used rabies virus tracing technology to map these inputs, but encountered non-specific tracing. To optimize this technology for a minor cell population (LepRb is not ubiquitously expressed in LH), we used LepRb-Cre mice and assessed how different titers of the avian tumor virus receptor A (TVA) helper virus affected rabies tracing efficiency and specificity. We found that rabies expression is dependent on TVA receptor expression, and that leakiness of TVA receptors is dependent on the titer of TVA virus used. We concluded that a titer of 1.0–3.0 × 10^7^ genomic copies per µl of the TVA virus is optimal for rabies tracing. Next, we successfully applied modified rabies virus tracing technology to map inputs to LepRb-expressing LH neurons. We discovered that other neurons in the LH itself, the periventricular hypothalamic nucleus (Pe), the posterior hypothalamic nucleus (PH), the bed nucleus of the stria terminalis (BNST), and the paraventricular hypothalamic nucleus (PVN) are the most prominent input areas to LepRb-expressing LH neurons.

## Introduction

Leptin is an adipocyte-derived cytokine and has a significant role in the regulation of body weight in humans as well as rodents^1–4^. Elevated leptin levels in the blood are associated with increased adiposity, which is a hallmark of obesity^5,6^. Leptin is known to increase energy expenditure, for example by increasing body temperature, and to decrease appetite and energy intake by action via its receptor, LepRb, which is expressed in a multitude of brain areas^1,2,7–9^. A brain area critically involved in the regulation of food intake which also expresses LepRb, is the lateral hypothalamus (LH)^10–12^. Only a subpopulation of neurons in the LH express LepRb and this population is distinct from for instance orexin producing neurons or melanin-concentrating hormone (MCH) neurons that do not express LepRb^13^. The LH relays information from neural circuits regulating energy balance to the mesolimbic system, such that a perceived negative energy balance results in motivation to initiate behaviours that result in energy intake. However, not all neuroanatomical inputs to LepRb-expressing LH neurons have been mapped.

**Figure 1:**
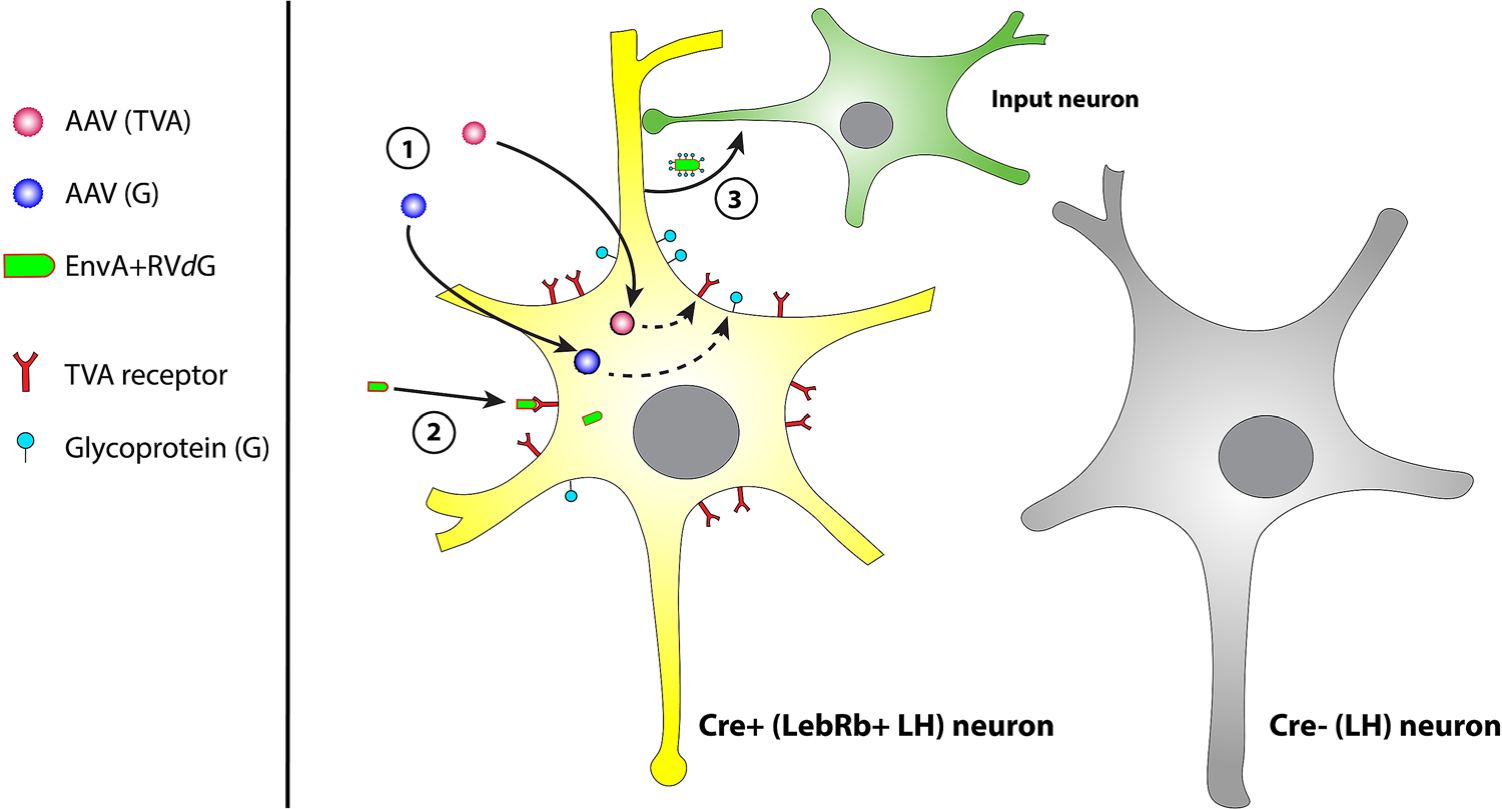
Working mechanism of modified rabies virus tracing technology. Modified rabies virus lacks the envelope protein G, which normally allows budding of new host cell-derived membrane-enveloped viral particles that have G on their membrane. Modified rabies is pseudotyped, so that it expresses EnvA as envelope protein. EnvA makes use of the avian-specific TVA receptor for entry in recipient cells. First (1), an injection with two helper AAV vectors delivers Cre-dependent TVA-mCherry and G in a mouse Cre-line. Only neurons that express Cre will start to express the TVA receptor and G. Second (2), when TVA and G are adequately expressed, mice are injected with modified G-deleted rabies virus (RV*d*G). EnvA-coated RV*d*G can only infect host-cells that express the TVA receptor and it needs host-cells to express G for *trans*-complementation in order to spread to presynaptic nerve terminals. Once EnvA-coated RV*d*G entered neurons via the TVA receptor, it replicates and uses G to obtain normal infectious properties. Thereby, it is able to spread monosynaptically to presynaptic nerve terminals. Since these input neurons do not express G, RV*d*G cannot infect subsequent nerve terminals from upstream input neurons. RV*d*G expresses GFP, allowing input mapping to Cre-positive neurons.

Since long, viral vectors have been used as methods of neuroanatomical tract tracing^14,15^. Modified rabies virus tracing is a relatively new tracing technique that can be used for qualitative and quantitative analysis of functional neuronal networks in the brain^14–26^. In modified G-deleted rabies virus (RV*d*G) rabies glycoprotein (G), an envelope protein essential for transneuronal transfer of rabies virus^18,27^, is deleted from the genome and replaced by green fluorescent protein (GFP). Therefore, RV*d*G needs host-cells to express G for *trans*-complementation in order to spread to presynaptic nerve terminals^18,27,28^. RV*d*G is pseudotyped with the avian ASLV type A (EnvA, Fig. 1), that makes use of the avian-specific TVA receptor for entry in recipient cells^18,27^. This restricts entry of RV*d*G to those cells that are genetically designed to express TVA. In the modified rabies virus tracing approach used in the current study, two helper adeno-associated viral vectors (AAV vectors) are injected simultaneously; one that carries TVA-mCherry which, once expressed in target neurons, allows EnvA-coated RV*d*G to enter, and one that carries rabies G which, once expressed in target neurons, leads to normal infectious properties of RV. To restrict input mapping to LepRb-expressing neurons, TVA and G are Cre-dependent^27,29^ and injected in LepRb-Cre mice, allowing TVA and G expression only in LepRb-expressing neurons. EnvA-coated RV*d*G will infect target neurons expressing TVA, being LepRb-expressing neurons, and subsequently spread monosynaptically to presynaptic nerve terminals, since only target neurons express G^27–29^. RV expresses GFP, which visualizes input neurons to LepRb-expressing neurons (Fig. 1).

Even though rabies tracing is a highly specific and effective tracing technique, to our knowledge little work has been done on the optimization of titers of the different vectors used. Previous studies mostly used control experiments to assess that rabies virus is dependent on expression of the helper viruses, and thus not leaky^19,28,29^. To the best of our knowledge, however, only one study has investigated the effect of different titers of Cre-dependent TVA on rabies efficiency and specificity^30^. This is crucial, in particular when only few Cre-positive neurons are present in a brain region, such as in the case of LepRb-expressing neurons in the LH. We noticed that during preparation of AAV viruses (when obtained from vector core facilities, as well as when grown in our own lab), a low amount of Cre-dependent genes is recombined in bacteria (in the absence of Cre-recombinase). Unspecific TVA receptor expression, a possible scenario when a high titer is used, undermines the specificity of RV tracing: little TVA receptor expression is already sufficient for EnvA-coated RV*d*G infection and subsequent retrograde spread of RV^23^. Leaky TVA receptor expression (expression in the absence of Cre-recombinase) and consequent unspecific RV spread cannot be distinguished from specific RV spread, thus compromising the specificity of tracing^23^. On the contrary, too little TVA expression will result in inefficient infection of EnvA-coated RV*d*G, thereby probably resulting in missed inputs. An optimal balance between specificity and efficiency of RV tracing is therefore a prerequisite. With the aim to optimize specificity and efficiency of RV tracing, we optimized the TVA titer and used this titer to map inputs to LepRb-expressing LH neurons. Mapping of these inputs will provide more insight into the organization of feeding behaviour-related neural-networks.

## Results

### Spontaneous TVA recombination in AAV plasmids

A quantitative real-time polymerase chain reaction (qPCR) was performed on the rAAV-Flex-TCB plasmid to estimate the amount of recombined TVA receptor DNA in a maxiprep, thus prior to AAV production and subsequent injection of the virus, and in the absence of added Cre recombinase. CT values of the qPCR on wPRE and unrecombined plasmid were used as guides to determine which concentration could be reliably detected. A CT value higher than 29 was regarded as non-specific (see Supplemental table 1; Negative row, wPRE column). wPRE was used as target sequence to amplify. A stock of wPRE containing plasmid was serially diluted to gerenate a standard curve. The presence of spontaneously recombined plasmid only becomes detectable by qPCR in the range of between 1.0 × 10^7^ genomic copies per µl (g.c./µl) and 1.0 × 10^8^ g.c./µl starting copies of plasmid (CT < 29, see Supplemental table 1). CT values at titers of 1.0 × 10^7^ g.c./µl and 1.0 × 10^8^ g.c./µl were 26,0 and 16,5 respectively. This indicates that the highest titer with nearly undetectable recombined versions of TVA DNA lies closer to 1.0 × 10^7^ g.c./µl than to 1.0 × 10^8^ g.c./µl. We estimated that a titer between 1.0 × 10^7^ g.c./µl and 3.0 × 10^7^ g.c./µl would be optimally specific and efficient. Delta CT at a titer of 1.0 × 10^7^ g.c./µl was 15.6.

### TVA expression is necessary for RVdG expression

**Figure 2:**
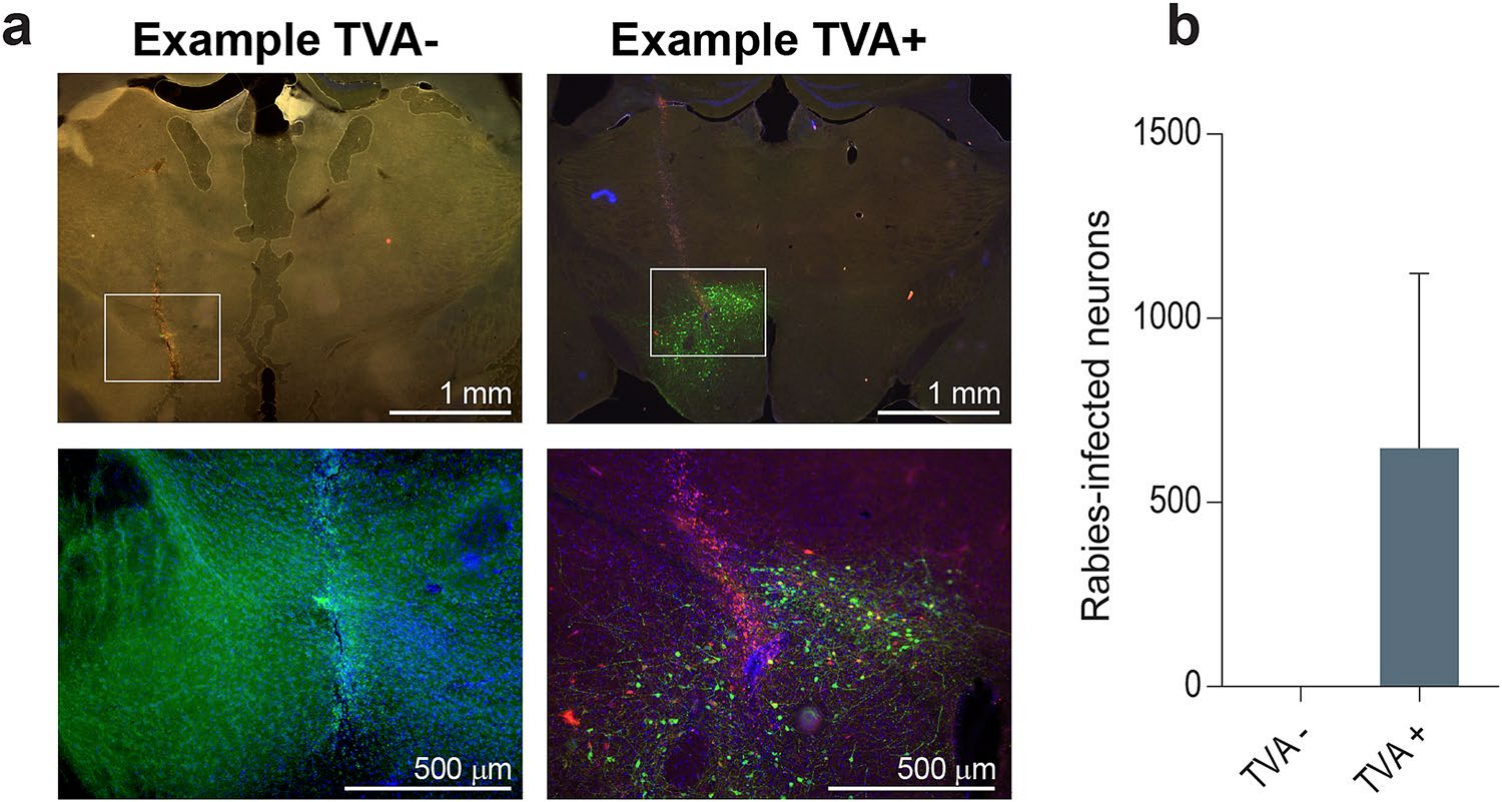
RV*d*G expression in C57BL6/J control mice with (TVA+) or without (TVA−) a prior injection of 1.0 × 10^9^ g.c./µl rAAV5-Flex-TCB. (**a**) Representative images of TVA− and TVA+ animals. Zooms are displayed under the original images. (**b**) TVA− mice expressed no RV*d*G (only background, thus non-specific GFP staining (likely autofluorescence from injection tract) is visible), while TVA+ mice expressed a remarkable higher level of RV*d*G. Initial version of graph was created using GraphPad Prism (version 7.0e).

Prior to assessing the effects of different TVA titers on efficiency and specificity of rabies tracing, we performed a control experiment to check if TVA expression is indeed necessary for RV*d*G expression to occur. Wildtype C57BL6/J mice were injected in the LH with 1.0 × 10^9^ g.c./µl G-deleted rabies virus, SAD-ΔG-eGFP(EnvA), with (TVA+; *n* = 2) or without (TVA−; *n* = 2) a prior injection of 1.0 × 10^9^ g.c./µl Cre-dependent TVA (rAAV5-Flex-TCB). In the absence of TVA, there was no expression and thus infection of RV*d*G. TVA-injected mice showed a high level of RV*d*G expression, whereasTVA-negative mice did not show clear specifically labelled neurons (Fig. 2), indicating that RV*d*G expression is dependent on TVA expression, but also that TVA receptor expression is leaky as there was expression observed in wild-type mice that do not express Cre.

### Leakiness of TVA receptor depends on titer

**Figure 3:**
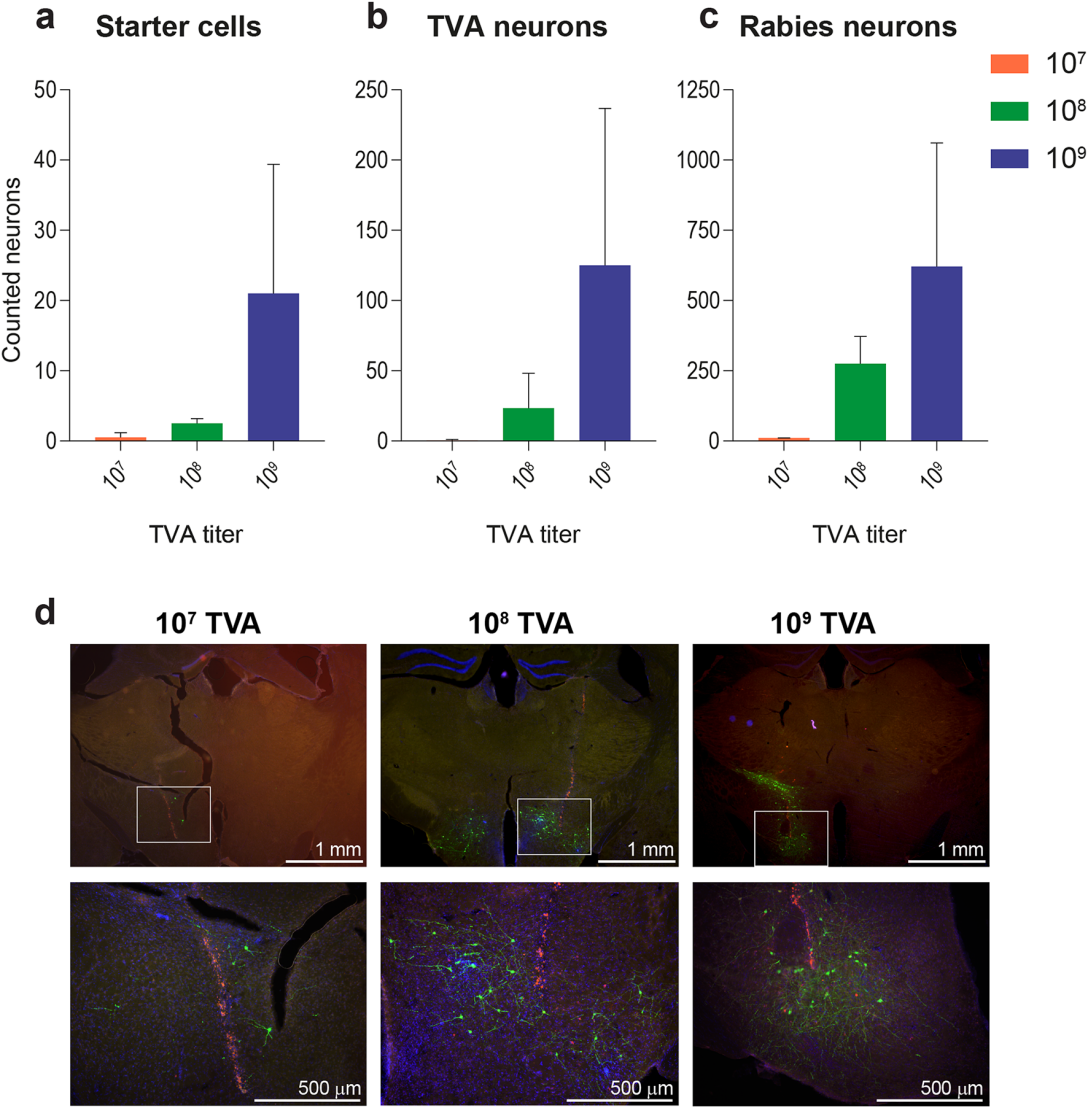
RV*d*G expression due to TVA leakiness depends on TVA titer. Number of starter cells (**a**), TVA-expressing neurons (**b**), and RV*dG*-expressing neurons (**c**) were quantified in C57BL6/J Cre-negative control mice that were injected with Cre-dependent TVA and G, followed by SAD-ΔG-eGFP(EnvA) in the LH. Number of starter cells [*F*(2,3) = 2.261, *p* = 0.252], TVA-expressing neurons[*F*(2,3) = 2.011, *p* = 0.279], and RV*dG*-expressing neurons [*F*(2,3) = 2.784, *p* = 0.207] showed a trend towards an increase with TVA titer, also visible in the representative images with zooms per titer underneath in d. For the 2.5 × images: 3.953 µm/pixel, and for the 10× zooms: 1.018 µm/pixel. Note that injections of 1.0 × 10^7^ g.c./µl and 1.0 × 10^8^ g.c./µl rAAV5-Flex-TCB were done bilaterally, whereas 1.0 × 10^9^ g.c./µl rAAV5-Flex-TCB was done unilaterally. Further, note that the y-axes are different for **a**, **b**, and **c**. Initial version of graphs in **a**, **b**, and **c** was created using GraphPad Prism (version 7.0e).

Many GFP-expressing neurons (M = 647, SD = 475.2 over all images) were detected in wildtype control mice that do not express Cre and that were injected with Cre-dependent TVA, rAAV5-Flex-TCB. This demonstrated that RV*d*G was able to infect neurons, which was likely due to TVA being expressed in the absence of Cre. This suggests that at this high titer of 1.0 × 10^9^ g.c./µl rAAV5-Flex-TCB, there is sufficient expression of TVA resulting in RV*d*G infection. Therefore, we reduced the titer of rAAV5-Flex-TCB to determine what the lowest titer of TVA was which still resulted in RV*d*G infection of LepRb-Cre mice, with neglectable infection in wildtype control mice. To this aim, wildtype C57BL6/J mice were injected with either 1.0 × 10^7^, 1.0 × 10^8^, or 1.0 × 10^9^ g.c./µl rAAV5-Flex-TCB in the LH. At least 21 days later these mice were injected with SAD-ΔG-eGFP(EnvA). Presence of starter cells (being mCherry and GFP positive), TVA positive cells (mCherry positive), and RV*d*G-infected cells (GFP positive cells) was quantified. Although relatively low numbers of starter cells (infected by both TVA and RV*d*G, Fig. 3a) and TVA-positive cells (Fig. 3b) were detected, a considerable trend towards an increase in number of both starter and TVA-infected neurons was found upon increasing TVA titer. Increasing TVA titer also increased the number of RV*d*G-infected cells (Fig. 3c). The numbers of RV*d*G-infected neurons were higher than the number of starter and TVA-positive cells for all tested titers, indicating that RV*d*G needs few starter cells to be able to infect and spread monosynaptically to input neurons. Figure 3d shows representative examples of immunohistochemical stainings of the LH of mice injected with the three different concentrations of rAAV5-Flex-TCB.

### Effect of different TVA titers on RVdG expression in LepRb-Cre mice

**Figure 4:**
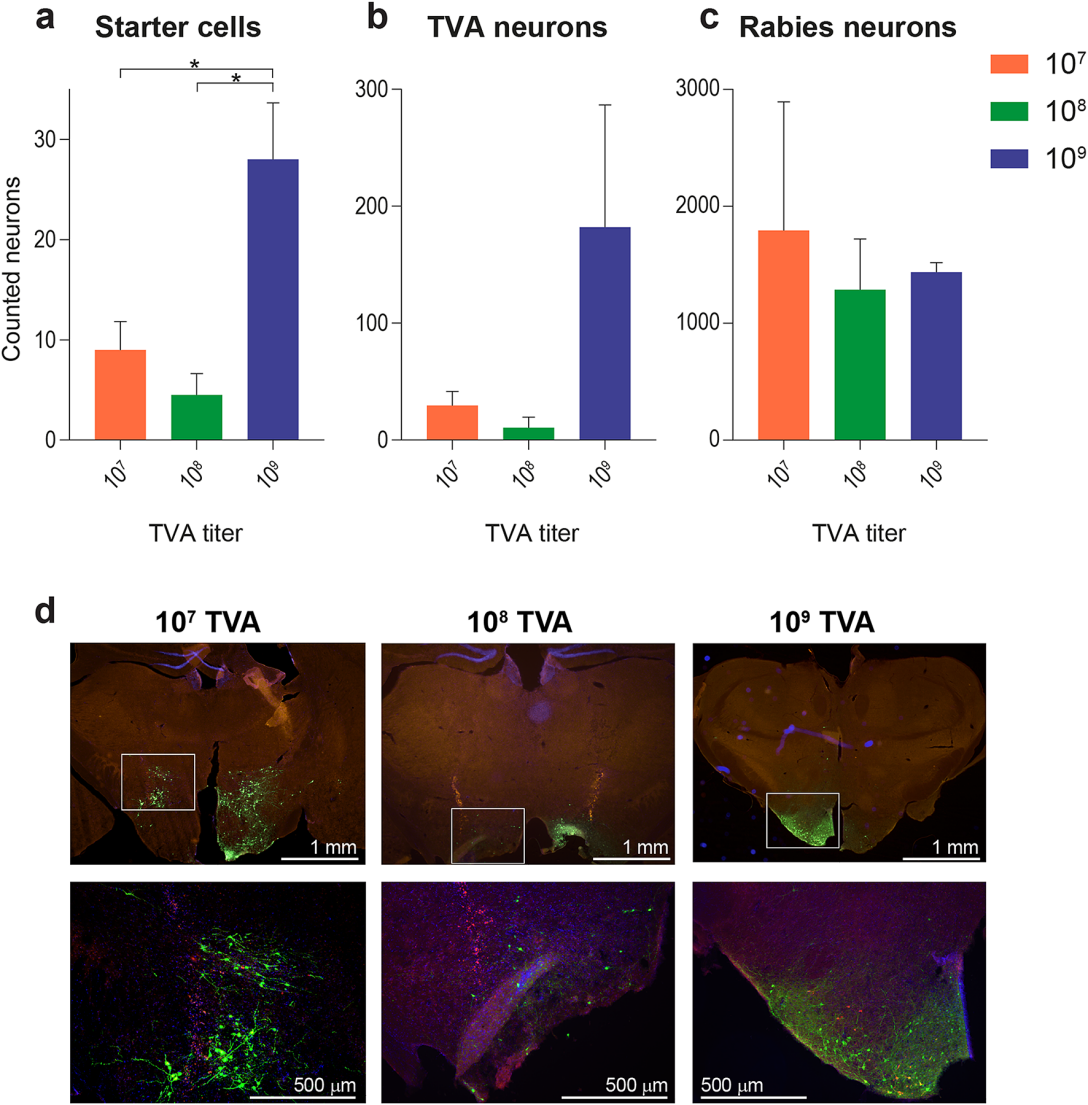
RV*d*G expression in LepRb-Cre mice. Number of starter cells (**a**), TVA-expressing neurons (**b**), and RV*dG*-expressing neurons (**c**) were quantified in LepRb-Cre mice that were injected with Cre-dependent TVA and G, followed by SAD-ΔG-eGFP(EnvA) in the LH. (**a**) Number of starter cells increased significantly with TVA titer [*F*(2,3) = 20.98, *p* = 0.017]. Post hoc comparison using Tukey HSD test showed that a TVA titer of 1 × 10^9^ g.c./µl [28 ± 5.66] resulted in significantly more starter cells than TVA titers of 1 × 10^7^ g.c./µl [9 ± 2.83] or 1 × 10^8^ g.c./µl [4.5 ± 2.12]. (**b**) TVA-expressing neurons showed a trend towards an increase with TVA titer [*F*(2,3) = 4.743, *p* = 0.118]. (**c**) No difference was found in the number of RV*dG*-expressing neurons [*F*(2,3) = 0.293, *p* = 0.765]. (**d**) These results are also visible in the representative images with zooms per titer underneath. For the 2.5 × images: 3.953 µm/pixel, and for the 10 × zooms: 1.018 µm/pixel. Note that injections of 1 × 10^7^ g.c./µl and 1 × 10^8^ g.c./µl rAAV5-Flex-TCB were done bilaterally, whereas 1 × 10^9^ g.c./µl rAAV5-Flex-TCB was done unilaterally. Further, note that the y-axes are ten-folds different for **a**, **b**, and **c**. Initial version of graphs in **a**, **b**, and **c** was created using GraphPad Prism (version 7.0e).

To assess the effect of different TVA titers on number of starter cells, TVA-expressing neurons, and RV*d*G-expressing neurons in the presence of Cre, LepRb-Cre mice were injected with either 1.0 × 10^7^, 1.0 × 10^8^, or 1.0 × 10^9^ g.c./µl rAAV5-Flex-TCB in the LH, followed by SAD-ΔG-eGFP(EnvA) at least 21 days later. Expression of starter cells, TVA-positive neurons, and RV*d*G-infected neurons was quantified. The amount of starter cells in the LH was significantly higher when 1.0 × 10^9^ g.c./µl rAAV5-Flex-TCB was injected as compared to the two lower titers (Fig. 4a), even though the injections with 1.0 × 10^9^ rAAV5-Flex-TCB were performed unilaterally and the injections with 1.0 × 10^7^ g.c./µl and 1.0 × 10^8^ g.c./µl rAAV5-Flex-TCB bilaterally. A trend in the same direction was found for TVA-positive cells (Fig. 4b). We found no significant differences between 1.0 × 10^7^ g.c./µl and 1.0 × 10^8^ g.c./µl rAAV5-Flex-TCB (Figs. 4a,b). No differences in RV*d*G expression were found between the different titers (Fig. 4c). Example images of the immunohistochemical sections display the same trends and are shown in Fig. 4d.

### Inputs to LepRb-expressing LH neurons

**Figure 5:**
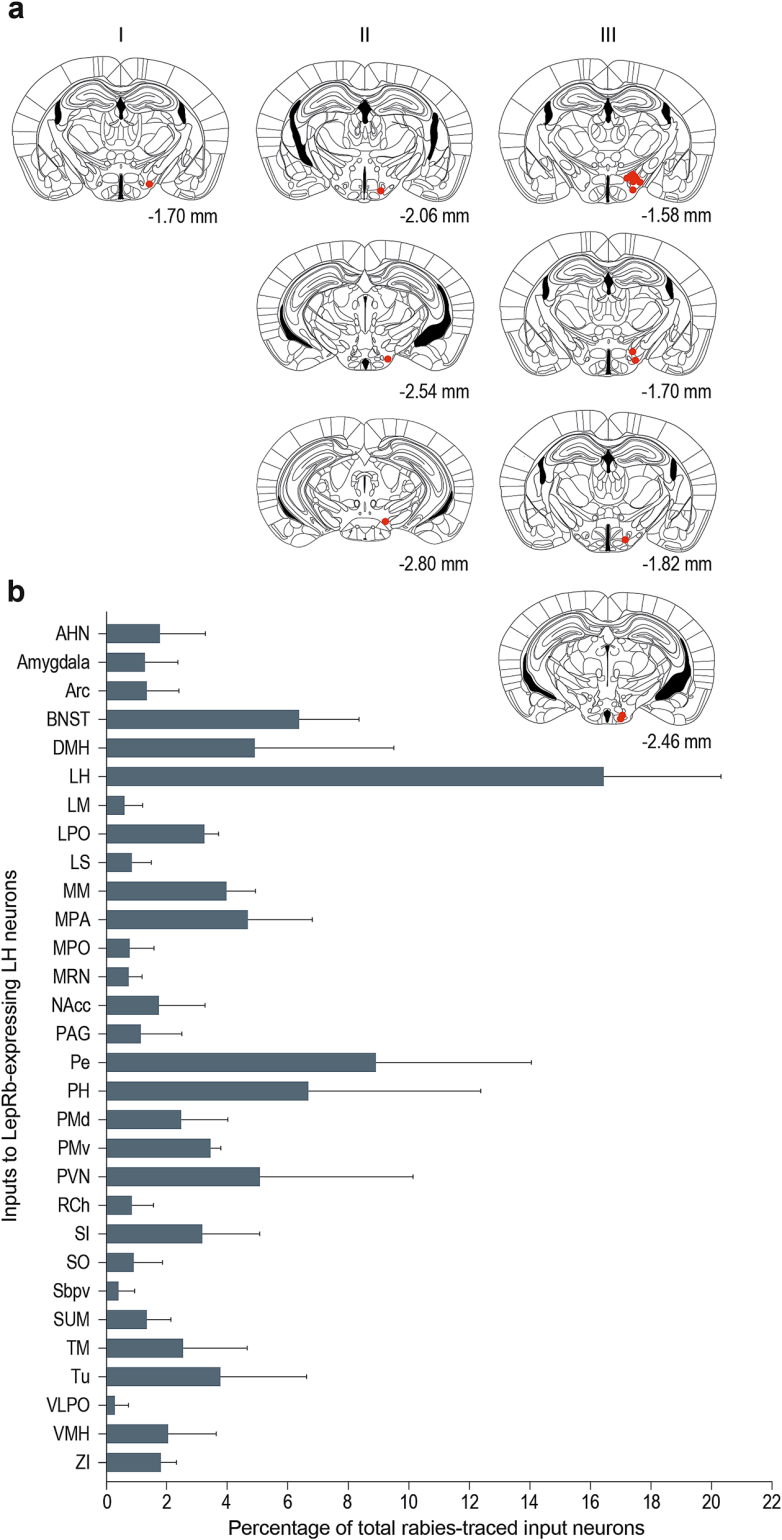
Rabies-traced inputs to LepRb-expressing LH neurons. (**a**) Starter cells found in the different mice that were used for input tracing to LepRb-expressing LH neurons. All mice were injected with 3.0 × 10^7^ g.c./µl rAAV5-Flex-TCB unilaterally. Mice in which the starter cells were not correctly targeted to the LH were excluded from the input analysis. (**b**) Different rabies-traced areas that input onto LepRb-expressing LH neurons. Areas were considered as input area, and displayed here, when they consisted of more than 1% of all rabies-traced inputs within one mouse. AHN, anterior hypothalamic nucleus; Arc, arcuate hypothalamic nucleus; BNST, bed nucleus of the stria terminalis; DMH, dorsomedial hypothalamic nucleus; LH, lateral hypothalamus; LM, lateral mammillary nucleus; LPO, lateral preoptic area; LS, lateral septal nucleus; MM, medial mammillary nucleus; MPA, medial preoptic area; MPO, medial preoptic nucleus; MRN, midbrain reticular nucleus; NAcc, nucleus accumbens; PAG, periaqueductal grey; Pe, periventricular hypothalamic nucleus; PH, posterior hypothalamic nucleus; PMd, premammillary nucleus dorsal part; PMv, premammillary nucleus ventral part; PVN, paraventricular hypothalamic nucleus; RCh, retrochiasmatic area; SI, substantia innominata; SO, supraoptic nucleus; Sbpv, subparaventricular zone; SUM, supramammillary nucleus; TM, tuberomammillary nucleus; Tu, tuberal nucleus; VLPO, ventrolateral preoptic nucleus; VMH, ventromedial hypothalamic nucleus; ZI, zona incerta. Initial version of graph in b was created using GraphPad Prism (version 7.0e).

Out of four LepRb-Cre mice that were injected with the titer found to be optimally specific and efficient in the qPCR experiment and other previously mentioned experiments, being 3.0 × 10^7^ g.c./µl rAAV5-Flex-TCB, three were correctly targeted to the LH. We quantified whole-brain RV*d*G expression to elucidate the different areas that input onto LepRb-expressing LH neurons (Fig. 5a). All areas that contained more than 1% of the total amount of input neurons per single mouse were considered input areas (Fig. 5b). We found that the LH itself, the periventricular hypothalamic nucleus (Pe), the posterior hypothalamic nucleus (PH), the bed nucleus of the stria terminalis (BNST), and the paraventricular hypothalamic nucleus (PVN) are the most prominent input areas to LepRb-expressing LH neurons.

## Discussion

We found that careful consideration of the titer of Cre-dependent TVA is of utmost importance to avoid non-specific results when using modified rabies virus tracing technology for mapping neuronal circuitries. Previous studies have shown that modified rabies virus tracing technology is a highly effective and specific method to map neuronal inputs to specific neurons in the brain^14,19,21,22,24,25^. We aimed to determine the most optimal titer of Cre-dependent TVA for mapping inputs to LepRb-expressing LH neurons, which still resulted in efficient rabies tracing of inputs in LepRb-Cre mice, but had neglectable infection in wildtype control mice.

Using qPCR we found that probably around 1 in every ~ 160,000 genomic copies of the TVA vector is already recombined during preperation of the plasmid, before injection; making a rough estimation by taking a difference of 3 CT values to be a log (10) difference in amount of starting DNA (CT values of wPRE increase with steps of ~ 3 between different sequential log dilutions), this resulted in 1 recombination in every ~ 160,000 genomic copies [log (10) of 15.6/3 = 158489]. This indicated that with an injection of 1 µl of the virus with a titer of 1.0 × 10^7^ g.c./µl, probably around 62,5 TVA particles will already be recombined and thus possibly be expressed in the absence of Cre recombinase. When a titer of 1.0 × 10^8^ g.c./µl is used probably around 625 TVA receptor particles will possibly be expressed in the absence of Cre, and with a titer of 1.0 × 10^9^ g.c./µl probably around 6250 TVA receptor particles will possibly be expressed. To achieve optimal specificity, it is therefore important to inject as little Cre-dependent, unrecombined, TVA particles as possible, so that as little as possible prematurely recombined TVA particles are injected. Of course this has its limitations, because efficiency must be optimal. However, not every viral particle results in infection. The TVA receptor DNA is packaged into AAV-particles which need a multiplicity of infection (MOI) of around 100.000 viral genomes per cell^31^, indicating that around 100.000 viral particles are needed to efficiently infect one neuron. Lower titers decrease the chance that all neurons will be infected. As there are approximately 100.000 neurons per mm^3^ and viral spread following an injection with a volume of 1 µl of 1.0 × 10^9^ g.c./µl is less than 1 mm^3^, injection of 1.0 × 10^9^ g.c. is sufficient to infect at least 10% of the neurons in such an volume^32,33^, assuming that indeed 10.000 neurons get infected with an injection of 1.0 × 10^9^ g.c. and an MOI of around 100.000 viral genomes per cell. Depending on the amount of targeted neurons, this would indicate that the amount of AAV-particles carrying a spontaneous recombined TVA receptor leading to infection is neglectable. However, MOIs refer to high efficiency of infection. For rAAV vectors^34^, the efficiency of an infectious particle to infect a cell is 0.2–1.0 × 10^–3^. When we use these assumptions and numbers based on literature and when there are 6250 infectious particles injected, one may expect 1–6 neurons to be injected, which is somewhat lower, but close to what we found.

A decrease in specificity due to leaky expression upon increasing TVA titer was also shown in vivo, where wildtype control mice that were injected with high titers of Cre-dependent TVA showed a remarkable high level of RV*d*G expression. This indicates that the TVA receptor is expressed leaky, thus in the absence of Cre recombinase. TVA leakiness, and thus RV*d*G expression, depends on titer, as RV*d*G expression increased with increasing titer. Still, LepRb-Cre mice showed a remarkable higher level of RV*d*G expression, compared with wildtype control mice (compare Figs. 3 and 4). Using qPCR we found that with a TVA titer of 1.0 × 10^9^ genomic copies per µl around 6250 TVA particles will be spontaneously recombined and may result in infection. Based on an MOI of 100.000 viral particles per cell^31^, 6250 would be neglectable. However, our in vivo results show that a TVA titer of 1.0 × 10^9^ genomic copies per µl indeed results in unspecific TVA infection. This is problematic for rabies tracing, especially when few Cre + neurons are present in the target area. Based on our qPCR and in vivo results we concluded that a titer of 3.0 × 10^7^ g.c./µl is probably the highest titer with lowest unspecific recombinations. Falsely RV*d*G-labelled input neurons due to leaky TVA receptor expression cannot be distinguished from ‘normal’ RV*d*G-labelled input neurons, and thereby leaky TVA expression hinders specific tracing^23^. RV*d*G expression itself was dependent on TVA, since wildtype control mice that were not injected with TVA did not express detectable levels of RV*d*G, consistent with previous studies^19,28,29^.

Based on our in vivo result that a titer of 1.0 × 10^7^ g.c./µl shows least expression in wildtype control mice, and on our qPCR result that a titer of 3.0 × 10^7^ g.c./µl is probably the highest titer with lowest unspecific recombinations, we considered a rAAV5-Flex-TCB titer between 1.0 and 3.0 × 10^7^ g.c./µl the most optimal titer for modified rabies traced input mapping of LepRb-expressing LH neurons. When Cre + neurons are present, a low titer of TVA receptors is already sufficient to infect Cre + neurons. A higher titer of TVA receptors seemingly leads to more starter cells in Cre + animals, but in fact it only hinders specificity, since it increases the chance of unspecific infection of Cre– neurons. By this means, a too high TVA titer results in many TVA-infected but Cre– neurons, especially in the case of few Cre + neurons. These unspecifically TVA-infected neurons subsequently become RV*d*G-labelled neurons and are then wrongly considered as input neurons. When many Cre + neurons are to be infected, this difference in specificity between low and high titers becomes neglectable. However, especially when there are fewer Cre + neurons it is important to be cautious with a too high TVA titer. Since LepRb-expressing neurons are not ubiquitously expressed in the LH, careful consideration is essential to avoid non-specific results.

Even though the highest titer of rAAV5-Flex-TCB tested, 1.0 × 10^9^ g.c./µl, resulted in more TVA-infected neurons (albeit unspecific), no differences in amount of RV*d*G-labelled neurons were found between the different TVA titers in Cre + animals. This is because RV*d*G is highly sensitive for TVA receptor expression^23^; RV*d*G only needs few starter cells to be able to infect and spread monosynaptically to input neurons. Thus, the seemingly high efficiency of the highest TVA titer was probably due to leakiness of the vector at this high concentration and does not affect efficiency of RV*d*G labelling.

Furthermore, we showed that, when the lower TVA titer is used for input mapping, many brain regions were found to provide input to LepRb-expressing LH neurons. The most prominent input areas to LepRb-expressing LH neurons are other neurons in the LH itself, the periventricular hypothalamic nucleus (Pe), the posterior hypothalamic nucleus (PH), the bed nucleus of the stria terminalis (BNST), and the paraventricular hypothalamic nucleus (PVN).

The LH is a central player in the regulation of energy balance, partly because it is a key area where leptin exerts its action upon^10,11,20,35–37^. The LH projects to the periventricular hypothalamic nucleus (Pe)^38^. The Pe also receives projections originating in the anterior hypothalamic area^39,40^, paraventricular nucleus of the hypothalamus^41^ and is densely innervated by ghrelin-containing projections^42^. A functional MRI study found a ghrelin induced response in the Pe^43^, underlining ghrelin sensitivity of the Pe and thereby its involvement in the regulation of feeding behaviour. The Pe is known to express the long-form leptin receptors^9^ and is now found to project to leptin sensitive LH neurons, thereby linking the working mechanisms of leptin and ghrelin.

The posterior hypothalamic nucleus (PH) plays an important role in autonomic and behavioural control mechanisms, and as such receives inputs from various brain regions, ranging from cortical and subcortical to brainstem and cerebellar regions^44^. The bed nucleus of the stria terminalis, the ventral pallidum, as well as the lateral and medial septal nuclei are some of the many input regions to the PH. Çavdar and colleagues suggested that these inputs areas, together with the PH, are all involved in autonomic regulation and make up different pathways that regulate autonomic and somatomotor activity^44^. Projections of the PH to the LH may therefore relay this autonomic information to the LH, where this autonomic information may be integrated with information about homeostatic energy balance, thus exerting an influence over feeding behaviour.

The bed nucleus of the stria terminalis (BNST) is innervated by ghrelin projections, just as the paraventricular hypothalamic nucleus (PVN), the Pe, the arcuate nucleus, the DMH and the LH^42^. It is already known to project to the anterior hypothalamic area^39,45^, and to relay cortico-limbic input to the PVN, where this information is integrated with visceral and interoceptive inputs^46^. The ventral part of the BNST sends glutamatergic and GABAergic efferents to the ventral tegmental area, stimulations of which results in aversive and anxiogenic behavioural phenotypes (for stimulation of glutamatergic projections) or rewarding and anxiolytic phenotypes (for stimulation of GABAergic projections)^47^. By this means, BNST circuit elements orchestrate divergent aspects of motivational and emotional processing^47^. Since we found that the BNST is a major input to LepRb-expressing LH neurons, this might indicate that the BNST provides motivational and emotional inputs to the regulation of feeding behaviour.

The paraventricular hypothalamic nucleus (PVN) is part of a circuit that is involved in metabolic regulation; it receives GABAergic projections from the LH that promote feeding^48^. The PVN is known to regulate pituitary gland function and feeding, it innervates autonomic preganglionic neurons, and is activated by intravenous leptin^45,49^. It is innervated by leptin-activated neurons in the caudal DMH, which may underlie the neuroendocrine and autonomic effects of leptin mediated by the PVN^45^. The PVN is also innervated by ghrelin-containing axon terminals^42^ and receives input from the arcuate nucleus of the hypothalamus, which are NPY and POMC neurons. Local ghrelin boutons in the PVN are in direct apposition to NPY axon terminals^42^. The fact that ghrelin produced in hypothalamic regions signals to the PVN, which is important for metabolic regulation, and that ghrelin synapses are anatomically close to NPY synapses, suggests that hypothalamic ghrelin is part of a central regulatory loop modulating the orexigenic drive. In the PVN, ghrelin increases the activity of NPY axon terminals, thereby probably increases the release of NPY and GABA, that modulate the activity of postsynaptic POMC neurons^42^. The PVN subsequently inputs to leptin sensitive neurons in the LH, thereby combining orexigenic information to neurons sensitive to anorexigenic signals. This underlines the idea that the LH is a central regulator of homeostatic regulation of energy balance.

The current study has some shortcomings. First of all, sample sizes were relatively small. Also, TVA injections with a titer of 1.0 × 10^7^ g.c./µl were done unilaterally, whereas injections with a titer of 1.0 × 10^7^ g.c./µl and 1.0 × 10^8^ g.c./µl were done bilaterally. This complicates comparison of the different titers, however, results were still elucidative since trends in clear directions were detected. Future studies should therefore perform experiments with a larger sample size in which all titers are injected unilaterally, so that intra- and interhemispheric inputs can be assessed within a single mouse, and that titers can be compared more easily.

Modified rabies virus tracing technology also has some limitations. Under most conditions, only a fraction of all inputs to the starter cells are labelled. This can be caused by insufficient expression of G in the starter cells, too few RV*d*G particles entering the starter cells, or a too short time available for trans-synaptic spread before starter cells die^27^. Secondly, since RV*d*G does not label all inputs, the probability of labelling might be different for different input cells. This might be caused by different numbers of synaptic contacts, the subcellular locations of synaptic contacts, or by different expression of receptors for G on axon terminals. This should always be considered when interpreting rabies tracing results^27^. Finally, RV*d*G-infected neurons have a surviving window of around 14 days, which is relatively long in comparison to other neurotropic virus-based tracing techniques, but which is still a short time to perform, for example, other studies to test RV*d*G-labelled neurons specifically. It is likely that cell health deteriorates before actual cell death; it has been suggested that cell viability is affected by 12 days after RV*d*G infection^50,51^. On the other hand, a timeframe of around 3–10 days is necessary to allow trans-synaptic spread of RV^27^, restricting the time window even further. We chose to euthanize animals 8 days after RV*d*G infection, since no differences in structural integrity of neurons or trans-synaptic efficiency could be detected at 8 days compared to 4 days post-infection^23^.

In summary, modified rabies virus tracing technology is a highly effective and specific method to elucidate neuronal circuits. In molecular neuroscience, novel viral vectors in combination with genetically-modified mice provide more sophisticated ways to map and manipulate specific neural circuits. Besides the Rabies tracing which we used here to map inputs to genetically identified neurons, the CANE technology allows to specifically map and target neurons that are activated following exposure to a certain challenge (Sakurai et al., Neuron 2016). As with CANE technology the TVA receptor is expressed under control of the fos promoter, the risk of leaky TVA expression from an AAV vector is circumvented. We place on cautionary note on the use of TVA in AAV vectors, because we found that specificity of rabies tracing decreases due to leaky TVA receptor expression when TVA titer increases. Since a titer between 1.0–3.0 × 10^7^ g.c./µl shows least expression in wildtype control mice and is efficient for mapping input neurons in LepRb-Cre mice, we recommend this titer as the most optimal rAAV5-Flex-TCB titer for mapping inputs to LepRb-expressing neurons in the LH. We furthermore showed that when a titer of 3.0 × 10^7^ g.c./µl is used for input mapping, LepRb-expressing LH neurons receive inputs from a broad variety of brain areas, but that neurons in the LH, the Pe, the PH, the BNST, and the PVN are the most prominent inputs. Thus, as a final note, before you go viral with modified rabies tracing technology, carefully consider the titer of TVA receptor you use.

## Methods

### Animals

Experiments were approved by the Animal Ethics Committee of the University Medical Center Utrecht, The Netherlands, were conducted in agreement with Dutch (‘Wet op de Dierproeven’, 2014) and European regulations (Guideline 86/609/EEC; Directive 2010/63/EU) and according to ARRIVE guidelines.

Healthy female and male wildtype C57BL6/J mice (n = 8) and transgenic C57BL6/J mice expressing Cre in leptin receptor-expressing neurons (LepRb-Cre mice; n = 12) were housed under controlled temperature and humidity conditions, with a 12 h-light/dark cycle (lights on at 7:00 a.m.). Based on their post-surgical condition, mice were housed in pairs or solitary. Animals had ad libitum access to water and standard chow, and tissues were provided as cage enrichment. Extra mashed chow was given in the home cage when animals showed signs of weakness after surgery. Mean (± standard deviation) body weight at time of first surgery was 27.0 (± 2.3) g.

### Quantitative real-time polymerase chain reaction

Quantitative real-time polymerase chain reaction (qPCR) was performed to determine the level of LoxP recombination of the rAAV-Flex-TCB (CAG-Flex-TCB, plasmid #48,332; Addgene.org) plasmid using a Quantstudio 6 qPCR instrument and the FastStart Universal SYBR Green mix (Roche). Reactions were performed on sequential log dilutions of the plasmid ranging from 1.0 × 10^9^ to 1.0 × 10^4^ genomic copies per µl, followed by a negative control. Reactions were performed with primer pairs specifically amplifying either the unrecombined, or the recombined version of the plasmid. A primer pair amplifying a segment of the wPRE element of the plasmid was used as a positive/internal control for each reaction. All reactions were performed in an end volume of 10 µl, containing 10 pmol forward and reverse primers, 2 µL genomic DNA template, and 5 µL FastStart SYBR Green mix. Annealing temperature was 60.0 °C. Primer sequences were as follows, all given 5’ to 3’: wPRE forward = CCCGTATGGCTTTCATTTTCTCC; wPRE reverse = CGGGCCACAACTCCTCATAA; non-recombined plasmid forward = AGCCGCGCCATGGTGGCGC; recombined plasmid forward = CGGCATGGACGAGCTGTACAAGTAA; (un)recombined common reverse = CTTTCACAAATTTTGTAATCCAGAGGTTGA. Cycle threshold (CT) values were obtained using the second derivative maximum method in the Quantstudio software. At the titer that resulted in detectable levels of recombined plasmid DNA, delta CT (dCT) was calculated by subtracting the CT value from the unrecombined version from the recombined version of the plasmid. From this dCT value the amount of recombined plasmids per number of genomic copies was estimated.

### Intracerebral viral vector injection

Mice were divided into eight groups; four groups (all n = 2) for the wildtype control mice and four groups (n = 2, n = 2, n = 4, n = 4) for the LepRb-Cre mice to assess the effect of different titers of TVA vector injection and to map inputs to LepRb-expressing LH neurons. For wildtype control mice, the first group received no TVA or glycoprotein (G) viral vector injection, thus only unilateral RV*d*G injection in the LH to assess necessity of the helper vectors for RV*d*G expression. The other three control groups all received different titers of the Cre-dependent improved TVA viral vector rAAV5-Flex-TCB, being 1.0 × 10^9^, 1.0 × 10^8^, and 1.0 × 10^7^ genomic copies (g.c.)/µl, and always the same titer of 0.9 × 10^9^ g.c./µl of the Cre-dependent G glycoprotein vector AAV8-CA-Flex-RG (plasmid #38,043; addgene.org). The first three LepRb-Cre groups received different titers of rAAV5-Flex-TCB, being 1.0 × 10^9^ (n = 4), 1.0 × 10^8^ (n = 2), and 1.0 × 10^7^ g.c./µl (n = 2), and always the same titer of 0.9 × 10^9^ g.c./µl of AAV8-CA-Flex-RG. In both the wildtype control and the LepRb-Cre groups unilateral injections were made when rAAV5-Flex-TCB was injected with a titer of 1.0 × 10^9^ g.c./µl, when rAAV5-Flex-TCB was injected with a titer of 1.0 × 10^8^ or 1.0 × 10^7^ g.c./µl bilateral injections were made. In the final LepRb-Cre group (n = 4) mice were unilaterally injected with 3.0 × 10^7^ g.c./µl of rAAV5-Flex-TCB and 0.9 × 10^9^ g.c./µl of AAV8-CA-Flex-RG.

Viral vector injections were performed as follows: before surgery mice received a subcutaneous injection with carprofen as analgesia (5 mg/kg, s.c., Carporal, AST Farma BV, the Netherlands) and were subsequently anesthetized by an intraperitoneal injection of a combination of 75 mg/kg ketamine (Narketan, Vetoquinol BV, the Netherlands) and 1 mg/kg medetomidine (SedaStart, AST Farma BV, The Netherlands). Xylocaine was sprayed on the skull for local anaesthesia (Lidocaine 100 mg/ml, AstraZeneca BV, the Netherlands). Using a stereotaxic apparatus (David Kopf Instruments, USA), 0.2 µl of a 1 to 1 mixture by volume of rAAV5-Flex-TCB (coupled to the red fluorophore mCherry; homemade from^23^) and AAV8-CA-Flex-RG (UNC Vector Core, USA) was injected into the lateral hypothalamus (LH; coordinates relative to Bregma: anteroposterior − 1.40 mm, mediolateral ± 1.80 mm at an angle of 10°, dorsoventral − 5.48 mm) at a speed of 0.1 µl/min. Depending on group assignment 0.2 µl of the mixture was injected unilateral or bilateral (0.2 µl per side). The injection needle was only retracted from the injection position after 10 min, to allow for diffusion of the vector mix into the tissue. Injections with G-deleted rabies virus pseudotyped with an avian virus envelope protein, called EnvA, (SAD-ΔG-eGFP(EnvA)^27,52^) were performed at least 21 days after the first surgery. 1.0 µl of 4.0 × 10^8^ focus forming units (ffu)/ml SAD-ΔG-eGFP(EnvA) was injected into the LH (using the same coordinates as before) at a rate of 0.2 µl/min. Again, the injection needle was kept in position for 10 min after injection, to allow for diffusion of the virus into the tissue. Atipamezole (2.5 mg/kg, SedaStop, AST Farma BV, the Netherlands) was given after surgery to reverse sedative effects. After surgery mice were carefully monitored and the two days following surgery carprofen (5 mg/kg, s.c., Carporal, AST Farma BV, The Netherlands) was administered to relieve post-operative pain.

### Tissue preparation

Exactly 8 days after RV*d*G injection mice were euthanized by intraperitoneal injection with an overdose of sodium pentobarbital (Euthanimal, Alfasan BV, The Netherlands) followed by intracardial perfusion-fixation with cold 0.1 M phosphate buffered saline (PBS), followed by 4% paraformaldehyde (PFA) in PBS. Brains were dissected and kept at 4 °C in PBS containing 4% PFA for at least 24 h for post-fixation. After post-fixation, brains were transferred to 30% sucrose with 0.1% NaN_3_ in PBS and kept at 4 °C, after at least another 48 h brains were frozen and sliced into 40 µm sections using a cryostat (Leica CM3050 S, Germany). Sections were collected as 1 in 6 series and stored in 30% sucrose with 0.1% NaN_3_ in PBS at 4 °C.

### Immunofluorescence

Sections were washed in PBS and blocked and permeabilized in PBS containing 5% normal goat serum, 5% normal donkey serum, and 0.25% Triton-X-100 for 1 h at room temperature. Sections were incubated overnight with the primary antibodies rabbit anti-dsRed (1:1000, cat # 632496, Clontech Laboratories, USA) to detect mCherry-fused TVA and chicken anti-GFP (1:1000, cat # ab13970, Abcam, UK) to detect RV*d*G in 2% normal goat serum and 0.1% Triton-X-100 in PBS at 4 °C. After washing in PBS, TVA-mCherry was visualized with Alexa-568-labeled goat anti-rabbit IgG (1:500, cat # ab175471, Abcam, UK) and RV*d*G was visualized with Alexa-488-labeled goat anti-chicken (1:500, cat # ab150169, Abcam, UK) in 2% normal goat serum and 0.1% Triton-X-100 in PBS. After incubation with secondary antibodies, sections were washed in PBS, incubated with DAPI (4',6-diamidino-2-phenylindole) to visualize cell nuclei, washed in PBS again, mounted and embedded in Fluorsave (Merck Millipore, the Netherlands).

### Image analysis

Immunofluorescent sections were photographed using a Zeiss Axio Scope A1 epifluorescent microscope (Carl Zeiss BV, Germany). Starter cells expressing both TVA and RV*d*G (expressing both fluorophores, thus yellow), TVA-positive neurons (red), and RV*d*G-positive neurons (green) were manually quantified in the entire brain using Fiji (version 1.0, ImageJ, National Institute of Health). Paxinos and Franklin mouse brain atlas was used as reference to identify brain areas expressing TVA and/or RV*d*G^53^. For input mapping, whole-brain inputs were only analysed if starter cells were discovered in the LH. For the last group of LepRb-Cre mice (n = 3), the Paxinos and Franklin mouse brain atlas^53^ was used as a reference for initial orientation. Slices were then matched to the corresponding image from the Allen Mouse Brain Atlas^54^ using Fiji, and input neurons were counted using the ‘multi-point’ function in Fiji. All input neurons were counted and ordered to create a list of brain regions that contained at least 1% of the total number of input neurons per mouse. Please note that for mapping input neurons, we only used data from bilaterally injected mice if starter cells were located in the same brain area.

### Exclusion criteria

Five mice were excluded (one wildtype control and four LepRb-Cre mice) since no starter cells—the initial TVA- and RV*d*G-infected cells—could be discovered in the LH.

### Data analysis

Statistical analysis was performed using GraphPad Prism (version 7.0e). Independent samples t-tests were used when appropriate. Numbers of starter cells, TVA-positive neurons, and RV*d*G-positive neurons were compared between different groups using a one-way analysis of variance (ANOVA) and data are shown as mean ± SD. When a significant effect was discovered, a post-hoc comparison was performed using a Tukey HSD test (*p* < 0.05).

## Supplementary Information

Supplementary Information.

## Data Availability

The data belonging to this manuscript is available upon specific request.

## References

[1] Friedman JM, Halaas JL (1998). Leptin and the regulation of body weight in mammals. Nature.

[2] Halaas, J. L. *et al.* Weight-Reducing Effects of the Plasma Protein Encoded by the Obese Gene. *Science (80-. ).* **269**, 543–546 (1995). 10.1126/science.7624777 7624777

[3] Rosenbaum M, Sy M, Pavlovich K, Leibel RL, Hirsch J (2008). Leptin reverses weight loss-induced changes in regional neural activity responses to visual food stimuli. J. Clin. Investig..

[4] Hinkle W, Cordell M, Leibel R, Rosenbaum M, Hirsch J (2013). Effects of reduced weight maintenance and leptin repletion on functional connectivity of the hypothalamus in obese humans. PLoS ONE.

[5] Slomp M (2019). Stressing the importance of choice—validity of a preclinical free-choice high-caloric diet paradigm to model behavioral, physiological, and molecular adaptations during human diet-induced obesity and metabolic dysfunction. J. Neuroendocrinol..

[6] Frederich RC (1995). Leptin levels reflect body lipid content in mice: evidence for diet-induced resistance to leptin action. Nat. Med..

[7] Davis JF (2012). Leptin regulates energy balance and motivation through action at distinct neural circuits. Biol. Psychiatry.

[8] Enriori PJ, Sinnayah P, Simonds SE, Garcia Rudaz C, Cowley MA (2011). Leptin action in the dorsomedial hypothalamus increases sympathetic tone to brown adipose tissue in spite of systemic leptin resistance. J. Neurosci..

[9] Elmquist JK (2001). Hypothalamic pathways underlying the endocrine, autonomic, and behavioral effects of leptin. Physiol. Behav..

[10] Elias CF (1999). Leptin differentially regulates NPY and POMC neurons projecting to the lateral hypothalamic area. Neuron.

[11] Leinninger GM (2009). Leptin acts via leptin receptor-expressing lateral hypothalamic neurons to modulate the mesolimbic dopamine system and suppress feeding. Cell Metab..

[12] de Vrind VAJ, Rozeboom A, Wolterink-Donselaar IG, Luijendijk-Berg MCM, Adan RAH (2019). Effects of GABA and leptin receptor-expressing neurons in the lateral hypothalamus on feeding, locomotion, and thermogenesis. Obesity.

[13] Burdakov D, Karnani MM, Gonzalez A (2013). Lateral hypothalamus as a sensor-regulator in respiratory and metabolic control. Physiol. Behav..

[14] Kuypers HGJM, Ugolini G (1990). Viruses as transneuronal tracers. Trends Neurosci..

[15] Vercelli A, Repici M, Garbossa D, Grimaldi A (2000). Recent techniques for tracing pathways in the central nervous system of developing and adult mammals. Brain Res. Bull..

[16] Ohara S (2009). Dual transneuronal tracing in the rat entorhinal-hippocampal circuit by intracerebral injection of recombinant rabies virus vectors. Front. Neuroanat..

[17] Ogawa SK, Watabe-Uchida M (2017). Organization of dopamine and serotonin system: Anatomical and functional mapping of monosynaptic inputs using rabies virus. Pharmacol. Biochem. Behav..

[18] Osakada F, Callaway EM (2013). Design and generation of recombinant rabies virus vectors. Nat. Protoc..

[19] Sun Y (2014). Cell-type-specific circuit connectivity of hippocampal CA1 revealed through cre-dependent rabies tracing. Cell Rep..

[20] Jennings JH, Rizzi G, Stamatakis AM, Ung RL, Stuber GD (2013). The inhibitory circuit architecture of the lateral hypothalamus orchestrates feeding. Science.

[21] Ruigrok TJH, Pijpers A, Goedknegt-Sabel E, Coulon P (2008). Multiple cerebellar zones are involved in the control of individual muscles: A retrograde transneuronal tracing study with rabies virus in the rat. Eur. J. Neurosci..

[22] Miyamichi K (2011). Cortical representations of olfactory input by trans-synaptic tracing. Nature.

[23] Miyamichi K (2013). Dissecting local circuits: parvalbumin interneurons underlie broad feedback control of olfactory bulb output. Neuron.

[24] Beier KT (2015). Circuit architecture of VTA dopamine neurons revealed by systematic input–output mapping. Cell.

[25] Watabe-Uchida M, Zhu L, Ogawa SK, Vamanrao A, Uchida N (2012). Whole-Brain Mapping of Direct Inputs to Midbrain Dopamine Neurons. Neuron.

[26] Grealish S (2015). Monosynaptic Tracing using Modified Rabies virus reveals early and extensive circuit integration of human embryonic stem cell-derived neurons. Stem Cell Rep..

[27] Callaway EM, Luo L (2015). Monosynaptic circuit tracing with glycoprotein-deleted rabies viruses. J. Neurosci..

[28] Wickersham IR (2007). Monosynaptic restriction of transsynaptic tracing from single, genetically targeted neurons. Neuron.

[29] Wall NR, Wickersham IR, Cetin A, De La Parra M, Callaway EM (2010). Monosynaptic circuit tracing in vivo through Cre-dependent targeting and complementation of modified rabies virus. Proc. Natl. Acad. Sci..

[30] Lavin TK, Jin L, Lea NE, Wickersham IR (2020). Monosynaptic tracing success depends critically on helper virus concentrations. Front. Synaptic Neurosci..

[31] Ellis BL (2013). A survey of ex vivo/in vitro transduction efficiency of mammalian primary cells and cell lines with Nine natural adeno-associated virus (AAV1-9) and one engineered adeno-associated virus serotype. Virol. J..

[32] Charvet CJ, Cahalane DJ, Finlay BL (2015). Systematic, cross-cortex variation in neuron numbers in rodents and primates. Cereb. Cortex.

[33] Herculano-Houzel S (2009). The human brain in numbers: a linearly scaled-up primate brain. Front. Hum. Neurosci..

[34] McCarty DM, Young SM, Samulski RJ (2004). Integration of Adeno-Associated Virus (AAV) and Recombinant AAV Vectors. Annu. Rev. Genet..

[35] Grossman SP, Dacey D, Halaris AE, Collier T, Routtenberg A (1978). Aphagia and adipsia after preferential destruction of nerve cell bodies in hypothalamus. Science.

[36] Sweeney P, Yang Y (2016). An inhibitory septum to lateral hypothalamus circuit that suppresses feeding. J. Neurosci..

[37] Stricker EM, Swerdloff AF, Zigmond MJ (1978). Intrahypothalamic injections of kainic acid produce feeding and drinking deficits in rats. Brain Res..

[38] Bjorklund, A., Hokfelt, T. & Swanson, L. W. The hypothalamus. In *Handbook of chemical neuroanatomy* 1–124 (Elsevier, 1987).

[39] Mitra A, Guèvremont G, Timofeeva E (2016). Stress and sucrose intake modulate neuronal activity in the anterior hypothalamic area in rats. PLoS ONE.

[40] Saper CB, Swanson LW, Cowan WM (1978). The efferent connections of the anterior hypothalamic area of the rat, cat and monkey. J. Comp. Neurol..

[41] Groenewegen HJ (1988). Organization of the afferent connections of the mediodorsal thalamic nucleus in the rat, related to the mediodorsal prefrontal topography. Neuroscience.

[42] Cowley MA (2003). The distribution and mechanism of action of ghrelin in the CNS demonstrates a novel hypothalamic circuit regulating energy homeostasis. Neuron.

[43] Wellman PJ (2011). Brain reinforcement system function is ghrelin dependent: studies in the rat using pharmacological fMRI and intracranial self-stimulation. Addict. Biol..

[44] Çavdar S (2001). The afferent connections of the posterior hypothalamic nucleus in the rat using horseradish peroxidase. J. Anat..

[45] Elmquist JK, Ahima RS, Elias CF, Flier JS, Saper CB (1998). Leptin activates distinct projections from the dorsomedial and ventromedial hypothalamic nuclei. Proc. Natl. Acad. Sci. USA.

[46] van Swieten MMH, Pandit R, Adan RH, van der Plasse G (2014). The neuroanatomical function of leptin in the hypothalamus. J. Chem. Neuroanat..

[47] Jennings JH (2013). Distinct extended amygdala circuits for divergent motivational states. Nature.

[48] Wu Z (2015). GABAergic projections from lateral hypothalamus to paraventricular hypothalamic nucleus promote feeding. J. Neurosci..

[49] Garza JC, Kim CS, Liu J, Zhang W, Lu X-Y (2008). Adeno-associated virus-mediated knockdown of melanocortin-4 receptor in the paraventricular nucleus of the hypothalamus promotes high-fat diet-induced hyperphagia and obesity. J. Endocrinol..

[50] Osakada F (2011). New rabies virus variants for monitoring and manipulating activity and gene expression in defined neural circuits. Neuron.

[51] Wickersham IR, Finke S, Conzelmann KK, Callaway EM (2007). Retrograde neuronal tracing with a deletion-mutant rabies virus. Nat. Methods.

[52] Callaway EM (2008). Transneuronal circuit tracing with neurotropic viruses. Curr. Opin. Neurobiol..

[53] Paxinos G, Franklin KBJ (2001). The Mouse Brain in Stereotaxic Coordinates.

[54] Allen Institute for Brain Science. Allen Mouse Brain Atlas. http://mouse.brain-map.org/ (2004).

