# Supplementary material for: Optimization of whole-brain rabies virus tracing technology for small cell populations
Source: Sci Rep. 2021 May 17;11:10400. doi: 10.1038/s41598-021-89862-5 (PMC8129069; doi:10.1038/s41598-021-89862-5)
Supplement: Supplementary file 1 — Supplementary Information. [file 41598_2021_89862_MOESM1_ESM.docx]

**SUPPLEMENTAL MATERIALS**

**Optimization of whole-brain rabies virus tracing technology for small cell populations**

**Theresia J.M. Roelofs^a,b^**

**Shanice Menting-Henry^a^**

**Lieke M. Gol^a^**

**Annelijn M. Speel^a^**

**Vera H. Wielenga^b^**

**Keith M. Garner^a^**

**Mieneke C.M. Luijendijk^a^**

**Alexandru A. Hennrich^d^**

**Karl-Klaus Conzelmann^d^**

**Roger A.H. Adan^a,c,^***

^a^Department of Translational Neuroscience, Brain Center, University Medical Center Utrecht, Universiteitsweg 100, 3584 CG Utrecht, the Netherlands

^b^Biomedical MR Imaging and Spectroscopy Group, Center for Image Sciences, University Medical Center Utrecht and Utrecht University, Bolognalaan 50, 3584 CJ Utrecht, the Netherlands

^c^Institute of Neuroscience and Physiology, The Sahlgrenska Academy at the University of Gothenburg, Sweden

^d^Max von Pettenkofer Institute Virology and Gene Center, Medical Faculty, Ludwig-Maximilians-University Munich, Munich 81377, Germany

***CORRESPONDING AUTHOR**

Roger A.H. Adan, PhD

Department of Translational Neuroscience, Brain Center Rudolf Magnus

University Medical Center Utrecht

Universiteitsweg 100, 3584 CG Utrecht, the Netherlands

Email: [r.a.h.adan@umcutrecht.nl](mailto:r.a.h.adan@umcutrecht.nl) ; Tel: (+31) 088-7568517

| *Supplemental table 1. CT values from qPCR with different primer pairs directed against the wPRE as a positive control, the unrecombined version of the plasmid, or the recombined version of the plasmid.* | | | |
| --- | --- | --- | --- |
| **TVA titer (genomic copies/µl)** | **wPRE** | **Unrecombined plasmid** | **Recombined plasmid** |
| *1,0×10^9^* | 5,8 | 5,1 | 12,5 |
| *1,0×10^8^* | 7,7 | 7,2 | 16,5 |
| *1,0×10^7^* | 10,8 | 10,4 | 26,0 |
| *1,0×10^6^* | 14,3 | 14,2 | 31,7 |
| *1,0×10^5^* | 17,3 | 17,5 | 32,1 |
| *1,0×10^4^* | 20,7 | 20,7 | 31,7 |
| *Negative* | 29,5 | 34,2 | 31,4 |
| *Cycle threshold (CT) values were obtained using the second derivative maximum method in the Quantstudio software.* | | | |

**
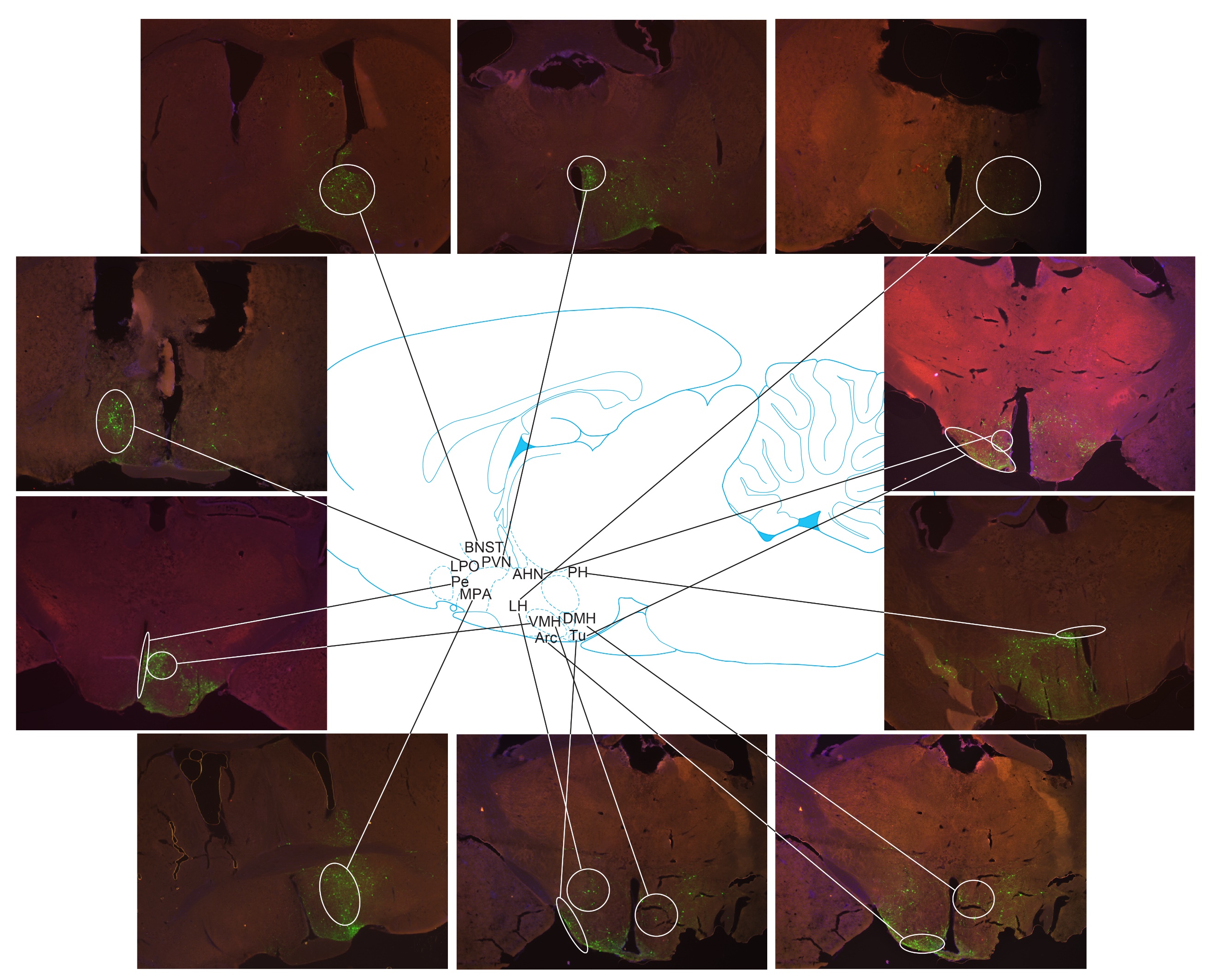
Supplemental Figure 1. Example images of input areas projecting to LepRb-expressing LH neurons.** A sagittal section of the rat brain (Template used with permission from Paxinos and Watson rat brain atlas, 6^th^ edition, 2007) is shown centrally in the figure. Example imput areas that send afferent projections to LepRb-expressing LH neurons are shown. The immunofluorescent images with input areas show the input neurons in green. AHN, anterior hypothalamic nucleus; BNST, bed nucleus of the stria terminalis; DMH, dorsomedial hypothalamic nucleus; LH, lateral hypothalamus; LPO, lateral preoptic area; MPA, medial preoptic area; MPO, medial preoptic nucleus; Pe, periventricular hypothalamic nucleus; PH, posterior hypothalamic nucleus; PVN, paraventricular hypothalamic nucleus; Tu, tuberal nucleus; VMH, ventromedial hypothalamic nucleus.
